# Incidentally vs non-incidentally diagnosed papillary thyroid carcinoma: are there differences?

**DOI:** 10.1530/ETJ-24-0106

**Published:** 2024-07-25

**Authors:** Inês Cosme, Ana Figueiredo, Sara Pinheiro, Valeriano Leite

**Affiliations:** 1 Department of Endocrinology, Unidade Local de Saúde Santa Maria, Lisbon, Portugal; 2 Department of Endocrinology, Instituto Português de Oncologia de Lisboa Francisco Gentil, Lisbon, Portugal

**Keywords:** incidental, non-incidental, papillary carcinoma, thyroid oncology

## Abstract

**Graphical abstract:**

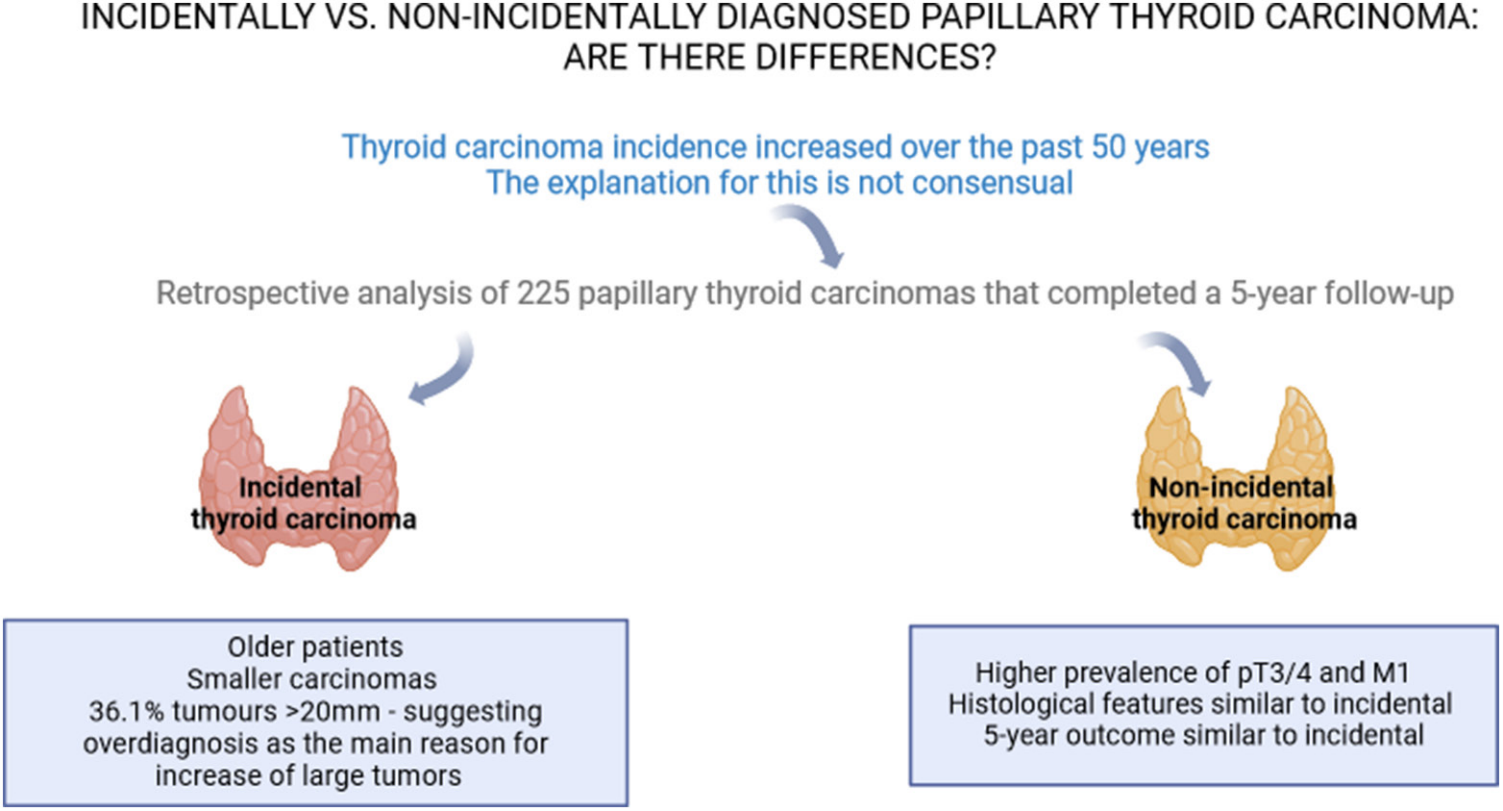

**Abstract:**

**Background**

Thyroid carcinoma (TC) incidence increased over the past 50 years. The explanation for this is not consensual.

**Objective**

Compare incidental vs non-incidental TC (ITC vs NITC) regarding demographic, clinical, histological data and 5-year clinical outcomes.

**Design**

Retrospective analysis of 225 papillary TC (PTC) cases that completed a 5-year follow-up.

**Methods**

Created 2 groups: ITC (including the incidentalomas) and NITC (cases of palpable or visible nodules or with thyroid compressive complaints).

**Results**

Included 225 PTC (122 were ITC). There were 95 women in ITC and 78 in NITC. ITC patients were significantly older (53.3 ± 14.8 vs 47.2 ± 17.7, *P* = 0.006). Groups had no differences in family history of TC. ITC mean tumour size was smaller (19.1 ± 9.2 vs 28.6 ± 16.2, *P* < 0.01). Tumours > 20 mm comprised 36.1% of ITC and 58.2% of NITC. We found no differences in tumour multifocality, histological thyroiditis, aggressive PTC subtypes, capsule or lymph-vascular invasion and gross extrathyroidal extension. There were no differences regarding the number of patients submitted to RAI or in RAI activity. pTMN staging showed higher prevalence of T3a and T4 cases (*P* < 0.01), and M1 status (*P* = 0.025) in NITC. There were no differences in the rates of persistence of disease. Logistic regression showed that the diagnostic modality had no impact on the 5-year clinical outcome.

**Conclusion**

ITC patients were older and had smaller tumours. NITC showed no worst histological features or 5-year clinical outcome. Approximately, one third of ITC had diameters > 20 mm. As even large tumours can be ITC, overdiagnosis is the most likely cause of increasing incidence of TC.

## Introduction

The incidence of thyroid carcinoma (TC) increased over the past 50 years (1, 2) mainly due to a disproportionate increase in small papillary TC (3). In 2020, the GLOBOCAN database reported worldwide 586,000 cases of TC (being the ninth highest cancer incidence) and 44,000 deaths related to TC (4, 5). The causes for this increasing incidence of TC diagnosis are controversial (6). The only known and well-established risk factors for TC are radiation exposure, mainly in childhood (7), and family history of TC (8). Traditionally, TC was detected by palpation of a neck nodule; however, nowadays, its detection by palpation is reported to account for only 30–40% of the diagnosed cases (1).

Some authors attribute the increasing incidence of TC to overdiagnosis which detects indolent lesions common in the general population and that will not be responsible for symptoms or death during patients’ lifespans. Overdiagnosis can be explained by the frequent use of imaging diagnostic tools such as ultrasound (US), computed tomography (CT), or ^18^F-fluorodeoxyglucose positron emission tomography (^18^F-FDG-PET), increased medical surveillance, and easier access to health care services (9, 10). However, other authors consider that overdiagnosis alone is unlikely to account entirely for the increased TC incidence (7, 11, 12), because a rising incidence has also been demonstrated for large thyroid tumors and tumors with clinically significant adverse features such as metastases and extrathyroidal extension (13). They claim that such a rise may be explained by other factors such as excessive body weight, greater height, hormonal exposures, and certain environmental pollutants (7). The aim of this study was to compare demographic, clinical, and histological data, pTNM classification and staging, radioactive iodine (RAI) treatment, postoperative thyroglobulin levels, and 5-year prognosis between incidental TC (ITC) and non-incidental TC (NITC).

## Materials and methods

Retrospective analysis of 225 consecutive patients who had undergone surgery for PTC and completed a 5-year follow-up at the Endocrinology outpatient clinic of *Instituto Português de Oncologia de Lisboa Francisco Gentil* (IPOLFG), in Portugal, from 2017 to 2018. Patients were categorized as incidentally or non-incidentally diagnosed. In the incidental group, we included patients whose TC was diagnosed during imaging workup (US, CT, or ^18^F FDG-PET) performed due to non-thyroidal diseases, thyroid dysfunction, routine check-up, symptoms not related to thyroid nodules, and TC diagnosed incidentally at the histological examination of benign thyroid lesions. In the non-incidental group, we included patients with palpable or visible thyroid nodules or with neck compressive complaints.

### Variables analysed

ITC and NITC groups were compared according to patients’ demographic data (sex and age at TC diagnosis), clinical data (TC family history and surgery type), TC histological features (size, multifocality, tumor subtype, invasion of tumor capsule, extrathyroidal extension, histological presence of thyroiditis), pTNM staging (14), treatment with RAI, postoperative thyroglobulin, risk of recurrence (14), and 5-year clinical outcome at which time patients were considered with biochemical evidence of disease (BED), indeterminate response, non-evidence of disease (NED) or structural evidence of disease (SED) (14).

As papillary aggressive subtypes, we considered diffuse sclerosing, tall cell, columnar cell, solid/trabecular, and hobnail (15). Gross extrathyroidal extension (gETE) was considered as macroscopic tumor invasion apparent on imaging or during surgery, and minimal extrathyroidal extension (mETE) was considered if only detected in the histological examination (14).

The postoperative thyroglobulin level was measured 4 to 6 weeks after surgery. Data were collected from the clinical records and pathological reports. The study was approved by the Ethics Committee of the IPOLFG. Informed consent was waived, according to the ethics committee's rules, since this was an observational retrospective study, and the database was anonymized.

### Statistical analysis

Quantitative variables were expressed as mean and standard deviation and categorical variables were expressed as frequencies. Comparisons among diagnostic groups were performed using the Chi-square test for categorical variables and the *t*-test for quantitative variables. Multivariate logistic regression was performed to analyze the impact of diagnostic modality, demographic, clinical, and histological features on the occurrence of NED at the 5-year evaluation. In the outcome persistence of disease, we included patients with BED, indeterminate response, and SED. *P* ≤ 0.05 was considered statistically significant. Statistical analysis was performed using SPSS v22 (IBM Statistics).

## Results

There were 225 patients diagnosed with PTC who completed a period of 5-year follow-up in the Endocrinology outpatient clinic of IPOLFG. This cohort includes 173 (76.9%) women, with a mean age at TC diagnosis of 50.1 ± 16.5 years. According to the diagnostic modality, patients were divided into two groups: ITC group, which included 122 patients (77.9% women), and the NITC group, which included 103 patients (75.7% women) (*P* = 0.704).

Out of those 225 patients, 122 (54.2%) were diagnosed incidentally: during imaging workups performed due to other diseases (*n* = 25), hypo/hyperthyroidism (*n* = 17), routine check-up (*n* = 31), symptoms not related to thyroid nodules (*n* = 36), or TC diagnosed incidentally at the histological examination of benign thyroid lesions (*n* = 13). The remaining 103 (45.8%) patients were diagnosed non-incidentally. Demographic, clinical, and histological data of the 225 patients with PTC are summarized in Tables 1 and 2.

**Table 1:** Demographic, clinical, and histological data of papillary thyroid cancer patients. Data are presented as *n* (%) or as mean ± s.d.

	Incidental group	Non-incidental group	*P*
*n*	122	103	
Age (years)	53.3 ± 14.8	47.2 ± 17.7	**0.006**
Female	95 (77.9)	78 (75.7)	0.704
TC family history	12 (9.8)	4 (3.9)	0.083
Total thyroidectomy	116 (95.1)	98 (95.1)	0.982
Mean tumour size (mm)^b^	19.1 ± 9.2 (7–58)	28.6 ± 16.2 (3–70)	**< 0.01**
Multifocality	69 (56.6)	51 (49.5)	0.291
Histological thyroiditis	23 (18.9)	14 (13.6)	0.289
Tumour capsule invasion	26 (21.3)	27(26.2)	0.388
Lymphovascular invasion	47 (38.5)	45 (43.7)	0.432
Gross extrathyroidal extension	11 (9)	15 (14.6)	0.195
Minimal extrathyroidal extension	44 (36.1)	14 (13.6)	**< 0.01**
Mean thyroglobulin after surgery (ng/mL)^a^	2.6±6.2	18.9 ± 112.3	0.11

^a^Irrespective of the surgery type; ^b^Values in parentheses are the range.

**Table 2:** Papillary thyroid cancer subtypes according to diagnostic groups. Data are presented as *n* (%).

PTC subtypes	Incidental group (*n* = 122)	Non-incidental group (*n* = 103)
Classic	73 (59.8)	54 (52.4)
Classic encapsulated	3 (2.5)	4 (3.9)
Follicular variant	33 (27.1)	31 (30.1)
Diffuse sclerosing	1 (0.8)	0 (0)
Tall cell	3 (2.5)	3 (2.9)
Clear cell	1 (0.8)	1 (1)
Oncocytic	2 (1.6)	4 (3.9)
Solid/trabecular	4 (3.3)	5 (4.9)
Whartin-like	2 (1.6)	1 (1)

Regarding aggressive PTC subtypes, there were 9 (7.4%) cases in the ITC group, and 10 (9.7%) cases in the NITC group (*P* = 0.531). During the follow-up, 88 (72.1%) patients of the ITC group received RAI treatment whereas RAI was applied to 79 (76.7%) of NITC patients (*P* = 0.435). The mean ± s.d . RAI activity used in the ITC group was 82.1 ± 37 mCi and in the NITC group was 98.4 ± 69.4 mCi (*P* = 0.065).

pTNM classification and staging are detailed in Table 3. Comparing the diagnostic groups, there were significant differences regarding T (*P* < 0.01) and M (*P* = 0.025) status but not in the N classification (*P* = 0.357).

**Table 3:** pTNM classification and staging according to diagnostic groups. Data are presented as *n* (%).

	Incidental group (*n* = 122)	Non-incidental group (*n* = 103)	*P*
**T**			**< 0.01**
T1a	17 (13.9)	14 (13.6)	
T1b	50 (41)	23 (22.3)	
T2	41 (33.6)	30 (29.1)	
T3a	3 (2.5)	21 (20.4)	
T3b	11 (9)	11 (10.7)	
T4a	0 (0)	3 (2.9)	
T4b	0 (0)	1 (1)	
N			0.357
Nx	73 (59.8)	52 (50.5)	
N0	15 (12.3)	11 (10.7)	
N1a	17 (13.9)	18 (17.5)	
N1b	17 (13.9)	22 (21.4)	
M			**0.025**
Mx	11 (9)	8 (7.8)	
M0	111 (91)	89 (86.4)	
M1	0 (0)	6 (5.8)	
pTNM staging			0.069
I	95 (85.6)	72 (75.8)	
II	16 (14.4)	18 (18.9)	
III	0 (0)	2 (2.1)	
IVb	0 (0)	3 (3.2)	
ATA recurrence risk			0.198
Low	37 (30.3)	35 (34)	
Intermediate	74 (60.7)	52 (50.5)	
High	11 (9)	16 (15.5)	

Values in bold indicate statistical significance.

The difference in staging between groups was not significant (*P* = 0.069), and there was no significant difference in the number of patients under 55 years in both groups (*P* = 0.085). There was also no difference in the risk of disease recurrence between groups, risk score (14). Tumor size distribution is detailed in Table 4.

**Table 4:** Tumor size distribution according to diagnostic groups.

	Incidental group (*n* = 122)	Non-incidental group (*n* = 103)	*P*
Tumour size (mm)			**< 0.01**
≤ 20	78 (63.9)	43 (41.7)	
> 20 to ≤ 40	41 (33.6)	37 (35.9)	
> 40	3 (2.5)	23 (22.3)	

Values in bold indicate statistical significance.

At the 5-year follow-up, there was persistence of disease in 47 (38.5%) patients of the ITC group, whereas in the NITC group, it was identified in 43 (41.7%) patients (*P* = 0.623) (Table 5).

**Table 5:** Five-year clinical outcome.

	Incidental group (*n* = 122)	Non-incidental group (*n* = 103)	*P*
**Outcome**			0.272
NED	75 (61.5)	60 (58.3)	
BED	5 (4.1)	5 (4.9)	
SED	5 (4.1)	11 (10.7)	
Indeterminate response	37 (30.3)	27 (26.2)	

BED, biochemical evidence of disease; NED, no evidence of disease; SED, structural evidence of disease.

Diagnostic modality in the ITC group was compared between patients with NED and disease persistence (DP) at follow-up. The diagnosis was made during imaging workup performed due to other diseases (*n* = 18, 24% in NED vs *n* = 7, 14.9% in DP), hypo/hyperthyroidism (*n* = 7, 9.3% in NED vs *n* = 10, 21.3% in DP), routine check-up (*n* = 17, 22.7% in NED vs *n* = 14, 29.8% in DP), symptoms not related to thyroid nodules (*n* = 25, 33.3% in NED vs *n* = 11, 23.4% in DP), or in histological examination of benign thyroid lesions (*n* = 8, 10.7% in NED vs *n* = 5, 10.6% in DP). There were no differences in the modality of detection between ITC NED and DP (*P* = 0.225).

Logistic regression analysis was performed to determine which demographic, clinical, and histological parameters were associated with NED at the 5-year evaluation. The results showed that the modality of diagnosis (incidental vs non-incidental) was not associated with NED (*P* = 0.824). Histological aggressiveness (*P* = 0.019), tumor multifocality (*P* = 0.021), and RAI activity (*P* = 0.024) had an impact on the 5-year clinical outcome and were associated with the defined outcome of persistence of disease.

## Discussion

TC incidence is increasing worldwide, and the *Surveillance, Epidemiology, and End ResultsDatabase* demonstrated an increase in diagnosis of TC of all sizes (11). The reason or reasons for this trend is (are) not consensual, and there are two possible theories: i) increased screening due to the widespread use of diagnostic imaging or ii) true increase in disease due to risk factors not yet entirely known (7, 16) such as obesity, nitrate fertilizers found in food, and smoking (11).

In our study, we included 225 PTC, from which 122 were diagnosed incidentally and the remaining in a non-incidental manner. We found differences concerning patients’ age at diagnosis, mean tumor size, mETE, and pM classification between the two groups.

Concerning age, similar to some authors, patients in the ITC group were older at the time of diagnosis (17, 18). Possibly, this is explained by the fact that the use of imaging exams increases with age, enabling the diagnosis of incidental TC. Considering age as a risk factor for malignant carcinomas, older patients with incidental TC may have a more aggressive disease (19).

Yoo *et al.* found a significant difference in sex distribution between ITC and NITC patients with a higher percentage of males in the ITC group (17). However, in our series, females predominated in both groups. In the NITC group, tumor size was larger than in ITC patients. This data contrasts with some studies (17, 20) but agrees with other reports (21, 22). Despite this, we found no difference in the proportion of microcarcinomas between the two groups, which also agrees with some of the published series (16, 22).

Specific histological features that can be associated with aggressiveness, such as multifocality, capsule and lymphovascular invasion, and lymph node metastasis, were similar between incidental and non-incidental TC groups. Interestingly, we found more cases of mETE in the incidentally diagnosed group. In addition, we identified a difference in the M status of pTNM classification between the two groups, with more cases of distant metastasis in the non-incidental diagnosed patients. Despite this, the number of patients in each pTNM stage was not different in both groups. Yoo *et al*. found a higher number of incidentally diagnosed patients in higher TNM stages compared to the non-incidental group; however, there were age differences that possibly interfered with staging (17).

In the evaluation performed 5 years after the diagnosis, we found no difference in the outcome between the two groups. These data suggest that the incidental group did not have a more indolent disease course compared to the non-incidental group. In addition, we also found that the diagnostic modality had no significant impact on the 5-year prognosis.

It has been suggested that a significant increase in the incidence of thyroid cancers with aggressive features, such as large tumor size, extra-thyroidal extension, and lymph node metastases, is against the theory that overdiagnosis is the cause for the rising incidence of TC since such tumors are less likely to remain clinically occult (13). However, an alternative hypothesis for such a rise is a more careful pathological examination and staging of TC patients, including a more frequent performance of central and lateral neck dissections.

Our study does have some limitations. Our institution is a tertiary cancer center, and our data may be biased by confounding factors that have not been identified. For instance, studies have shown that incidentally detected thyroid carcinomas account for 11% to 15% of thyroid malignant carcinomas (21, 23), and in our work, ITC cases comprised 54.2% of the cases. However, in South Korea, Li *et al.* (24) suggested that between 2008 and 2012, 90% of cases of thyroid cancer in Korean women were due to overdiagnosis. In our work, performed in a country where thyroid cancer was in 2020 the fourth most prevalent cancer in females (25), suggesting a high prevalence of overdiagnosis, ITC cases comprised 54.2% of the cases.

The strengths of this study are the examination of consecutive patients with a PTC diagnosis in a single institution following the same protocols, with all incidental carcinomas being included in the analysis. In conclusion, ITC patients were older and had smaller tumors than NITC. However, approximately one-third of ITC patients had tumor diameters above 20 mm, suggesting that even large tumors can be detected incidentally, a finding that suggests overdiagnosis as the most likely cause for the significant rise in the incidence of thyroid cancer.

## Declaration of interest

The authors declare that there is no conflict of interest that could be perceived as prejudicing the impartiality of the study reported.

## Funding

This work did not receive any specific grant from any funding agency in the public, commercial, or not-for-profit sector.

## Author contribution statement

IC wrote the manuscript. AF, SP, and VL conceived the study and reviewed the manuscript. The graphical abstract was created using BioRender.
